# Robotic posterior retroperitoneoscopic adrenalectomy: initial experience with Hugo™ RAS system

**DOI:** 10.1007/s11701-025-02414-1

**Published:** 2025-06-16

**Authors:** Martí Manyalich-Blasi, David Saavedra-Pérez, Leidy M. Fajardo Guzman, Maria Magdalena Llompart, Jordi Ardid Brito, Juan Jose Espert, Antonio Rull Ortuño, Miguel Pera Roman, Oscar Vidal

**Affiliations:** 1 Service of General and Digestive Surgery, Institute Clinic of Digestive and Metabolic Diseases (ICMDM), Hospital Clinic de Barcelona, Universitat de Barcelona, Villarroel 170, Escalera 10, Planta 3., 08036 Barcelona, Spain; 2 General and Digestive Surgery Department, Hospital General de la Plaza de la Salud, Santo Domingo, República Dominicana; 3 General and Digestive Surgery Department, Institut d’Investigacions Biomediques August Pi i Sunyer (IDIBAPS), Hospital Clinic de Barcelona, Universitat de Barcelona, Barcelona, Spain

**Keywords:** Robot-assisted surgery, Adrenalectomy, Robotic retroperitoneal adrenalectomy, RAS Hugo™ platform

## Abstract

Robot-assisted surgery has revolutionized minimally invasive procedures, offering superior three-dimensional visualization and mobile instruments suitable for smaller areas. For this reason, robotic retroperitoneal adrenalectomy (RRA) is emerging as an ideal procedure for this technology. This study aimed to assess the outcomes of the first 10 consecutive cases of this procedure using the RAS Hugo™ platform. Conducted between July 2023 and February 2024, the surgeries were performed with patients in the prone position, accessing the retroperitoneal space using standard endoscopic techniques. High-energy sealing instruments were utilized for adrenal vein sectioning, and specimens were extracted using protective bags. Ten surgical interventions were performed, with six male patients and four female patients. Most patients underwent surgery due to suspected primary hyperaldosteronism (*n*=7), while the remainder were operated on for Cushing’s syndrome (*n*=3). Median patient age was 58 years (range 50–73) with a median BMI of 28.5 kg/m^2^ (range 21–36), and American Society of Anaesthesiologists (ASA) risk scores were evenly split between ASA II and ASA III. Lesions were equally distributed between the right and left adrenal glands, with a median tumor size of 1.5 cm (range 0.5–3.5). Median operative time was 124.5 min (range 102–198), with one case requiring conversion to endoscopic approach due to pyelonephritis. No postoperative complications were reported, and median hospital stay was 1 day (range 1–3). RRA demonstrates feasibility for selected patients, offering enhanced image resolution and precision in confined spaces. However, challenges such as increased operative time and the need for skilled teams warrant consideration.

## Introduction

Posterior retroperitoneoscopic adrenalectomy (PRA) is a surgical technique introduced in 1994 and developed by Prof. Martin Walz in 2001. PRA offers advantages over laparoscopic approaches by avoiding entry into the peritoneal cavity, thereby reducing the risk of intra-abdominal injuries. Surgeons performing PRA must be familiar with the posterior anatomical perspective, as the procedure involves accessing the perirenal fascia and the posterior surfaces of the kidney and adrenal gland through a posterior approach [1].

The evolution of adrenal surgery towards minimally invasive techniques focuses particularly on laparoscopic adrenalectomy (LA) as the gold standard for treating small- to medium-sized benign adrenal tumors due to its advantages such as shorter hospital stays and lower morbidity rates. This is where the emergence of alternative approaches like PRA, specifically the posterior retroperitoneal endoscopic approach popularized by Walz et al., stands out, offering a viable alternative to the transperitoneal laparoscopic method [1, 2].

Adrenal diseases, including pheochromocytomas and other functional and non-functional tumors, pose a clinical challenge due to their complexity and potential for serious complications. RRA represents a significant advancement in the field of endocrine surgery, merging the precision of robotic technology with the accessibility and benefits of the retroperitoneal approach.

Piazza et al. in Italy carried out the initial use of robotic technology in adrenal gland surgery in 1999 [3]. Berber et al. described in 2010 for the first time the technique for RRA. They demonstrated the feasibility and safety of this approach in an initial series of eight patients [4]. Since then, RRA has emerged as an innovative surgical technique in managing adrenal pathologies, promising precision and efficacy in clinical practice.

In this context, this study focuses on evaluating the perioperative outcomes of the first 10 consecutive cases of RRA using the RAS Hugo™ platform. Analyzing RRA as a surgical procedure, clinical results, and lessons learned at the Hospital Clínic of Barcelona, we highlight its ideal setting for investigating and refining this advanced technique, leveraging its experience in minimally invasive surgery and commitment to excellence in endocrine disease treatment.

Through studying our initial experience, we aim to identify the benefits and challenges associated with adopting RRA and provide recommendations for its effective implementation in clinical practice. Through a multidisciplinary and collaborative approach, we hope to contribute to the continuous advancement of this surgical modality and improve outcomes for our patients.

## Methods

### Study design

This study consisted of a consecutive case series of patients who underwent RRA at the Hospital Clínic of Barcelona. This retrospective review analyzed previous cases of RRA, considering demographic variations, preoperative diagnoses, presence of comorbidities, and occurrence of complications to evaluate their postoperative evolution.

### Participants

Adult patients diagnosed with benign or malignant adrenal gland pathologies who were candidates for RRA, operated on during the period from July 2023 to February 2024, were included in this study.

### Data collection procedure

For this prospective study, we reviewed the medical records of patients treated with RRA at the Hospital Clínic of Barcelona since the implementation of the technique. Collected data included demographic information, preoperative diagnoses, surgical details, perioperative complications, and long-term outcomes.

### Surgical technique

Robotic retroperitoneal adrenalectomies were performed using the RAS Hugo robotic system. Standard surgical protocols established by the surgical team at the Hospital Clínic of Barcelona were followed, with adjustments made as necessary. Technical details of each procedure, including surgery duration, estimated blood loss, and intraoperative findings, were recorded.

Posterior RRA is a surgical technique recognized for its ease of learning, with a learning curve estimated between 20 and 40 procedures according to the European Society of Endocrine Surgeons. Previous experience of the surgeon in laparoscopy may shorten this curve [5].

### Overview of the technique

Here, we describe the ARR technique in ten standardized steps, including operating table setup, necessary instruments, and surgical team coordination. Constant communication among the surgeon, assistant, instrument nurse, and anaesthesiologist is crucial for patient safety and surgical success [6].

#### Ten standardized steps of PRA

1. Patient positioning
2. Placement of initial trocars
3. Creation of the workspace
4. Placement of the third trocar
5. Identification of the upper pole of the kidney
6. Identification of the inferior vena cava (IVC)
7. Dissection and ligation of the adrenal vein
8. Dissection of the entire gland
9. Extraction with an extraction bag
10. Final review (hemostasis) and closure

Surgical instruments and operating table setup

o Instruments: common robotic materials, including a 30° camera and three trocars (11 mm optic trocar and two 8 mm assist trocar), a non-traumatic clamp, and an energy device like LigaSure®.
o Operating table setup: the patient is placed in the prone position with rollers under the iliac crests and thorax, allowing the abdominal viscera to move away from the retroperitoneum. The table should support hip flexion close to 90° with bent and supported knees.

### Surgical team

The surgical team includes the surgeon, first assistant, and instrument nurse. Proper placement and coordination among team members are crucial for successful PRA.

### Detailed steps

1. Patient positioning
  Place the patient in a prone position with arms positioned above the head and at the sides.
  Position rolls under the iliac crests and thorax to allow abdominal viscera to move away from the retroperitoneum.
  Ensure that the thighs are nearly in a 90° flexion position with knees bent and supported.
2. Placement of Initial Trocars (Optic Trocar and Posterior Axillary Trocar)
  Make an approximately 1.5 cm skin incision below the tip of the 12th rib for the robotic optic trocar 11 mm.
  Place the 8 mm posterior axillary trocar below the tip of the 11th rib.
3. Creation of the workspace
  Use a 30° camera in the balloon trocar and create the workspace by pulling down the perirenal fat and opening the perirenal fascia.
4. Placement of the third trocar (medial trocar)
  Place the third trocar (8 mm) paravertebral, between the spine and the optic trocar at an acute angle toward the adrenal gland.
5. H RAS Hugo™ platform docking
  After the placement of the 3r trocar, the robotic arms’ docking is performed as shown in Fig. 1.

**Fig. 1:**
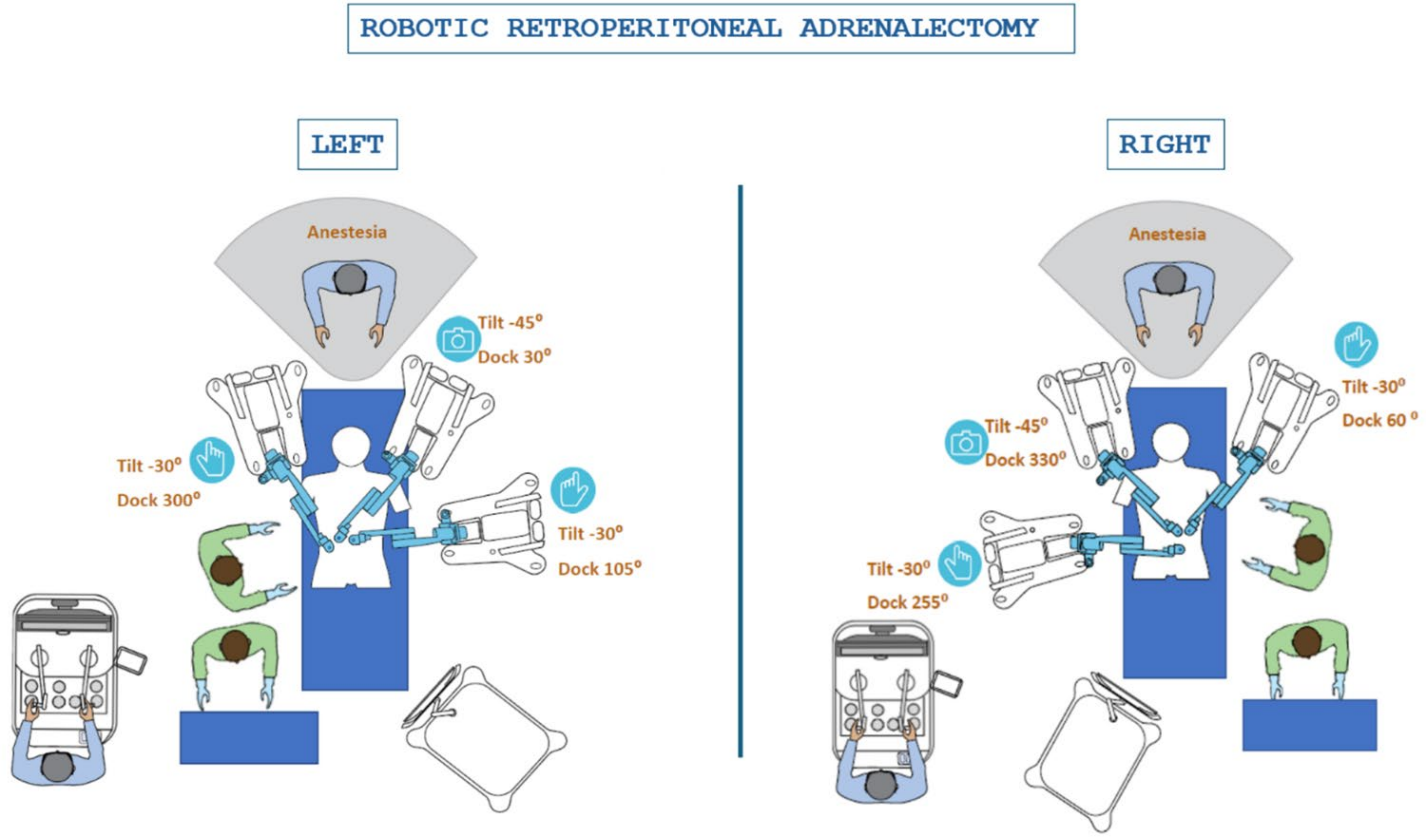
Robotic retroperitoneal adrenalectomy
  Place the third trocar (8 mm) between the spine and the balloon trocar at an acute angle toward the adrenal gland.
6. Identification of the upper pole of the kidney
  Dissect from lateral to medial and from bottom to top to find the upper pole of the kidney, providing the necessary anatomical reference point for further dissection.
7. Identification of the inferior vena cava (IVC)
  Identify and dissect the posterior wall of the IVC to expose the adrenal vein.
8. Dissection and ligation of the adrenal vein
  On the right side, the adrenal vein is located in a postero-medial position. On the left side, it drains into the phrenic vein and the left renal vein.
9. Dissection of the entire gland
  Use blunt dissection and energy devices to separate the gland from surrounding structures and ligate the remaining arteries.
10. Extraction with an extraction bag
  Place the gland in an extraction bag, remove it, and reintroduce the balloon trocar if necessary.
11. Final review (Haemostasis) and closure
  Ensure hemostasis by irrigating the surgical field and checking for absence of bleeding. Close the aponeurosis and suture the skin.

## Tips and tricks

o Proper trocar placement is critical.
o High pneumoretroperitoneum pressures (20-25 mmHg) help create ample workspace.
o Precise anatomical knowledge and attention to detail can significantly improve surgical efficiency and safety.

### Postoperative care

Patients can resume oral intake in the afternoon and be discharged the next day with analgesics. For patients with pheochromocytoma, intensive monitoring is recommended due to the risk of hemodynamic complications. Follow-up includes biochemical tests 2–4 weeks after surgery and lifelong monitoring [6].

### Final notes

Standardizing the ARR technique in ten steps facilitates learning. Surgeons should adapt to intraoperative challenges and rely on their laparoscopic skills acquired in other procedures to effectively navigate the learning curve.

### Outcome assessment

Primary outcome measures included conversion rates to laparoscopy or open surgery, complete lesion resection rates, and perioperative complication rates. Secondary outcomes included length of hospital stay, postoperative recovery, and disease recurrence rates.

### Statistical analysis

Descriptive data analysis was conducted to summarize patient characteristics and surgical outcomes. Appropriate statistical tests were used to compare results between retrospective groups and to identify factors associated with surgical outcomes.

## Results

### Demographic and clinical characteristics

This study included 10 patients undergoing RRA between July 2023 and February 2024. Of these patients, 6 (60%) were male and 4 (40%) were female, with a median age of 58 years (range 50–73 years). The mean body mass index (BMI) was 28.5 kg/m^2^ (range 21–36.7 kg/m^2^). According to the ASA anesthesia risk classification, 50% of the patients were classified as ASA II and the other 50% as ASA III. Table 1 presents the demographic and clinical data of the studied patients.

**Table 1:** Demographic and clinical characteristics

Demographic and clinical data	Number	Percentage
Number of patients	10	100
Sex		
Male	6	60
Female	4	40
Age (years)		
50–59	3	30
60–69	5	50
70–79	2	20
BMI (kg/m^2^)		
18.5–24.9	3	30
25.0–29.9	4	40
30.0–34.9	2	20
35.0–39.9	1	10
ASA		
II	5	50
III	5	50

Source: Data collected from digital clinical records of patients undergoing robotic retroperitoneal adrenalectomy at Hospital Clínic de Barcelona, during the period July 2023 to February 2024.

### Diagnoses and surgical characteristics

The majority of patients underwent surgery due to suspected primary hyperaldosteronism, accounting for seven cases (70%), while the remaining three cases (30%) were operated on for Cushing’s syndrome. The laterality of the lesion was evenly distributed, with 50% of cases affecting the right adrenal gland and 50% affecting the left adrenal gland. The tumor sizes had a median of 1.5 cm (range 0.5–3.5 cm). The diagnoses and surgical characteristics are presented below in Table 2.

**Table 2:** Diagnoses and surgical characteristics

	Number of cases	Percentage
Diagnose		
Primary hyperaldosteronism	7	70%
Cushing’s syndrome	3	30%
Laterality of the lesion		
Right adrenal	5	50%
Left adrenal	5	50%
Tumour size (cm)		
< 1 cm	4	40%
1-2 cm	4	40%
> 2 cm	2	20%

Source: Data collected from digital clinical records of patients undergoing robotic retroperitoneal adrenalectomy at Hospital Clínic de Barcelona, during the period July 2023 to February 2024.

### Surgical and postoperative outcomes

The median operative time was 124.5 min (range 102–198 min). Only one patient (10%) required conversion to laparoscopy due to inflammation from pyelonephritis. Postoperative complications were not reported in 9 out of 10 patients (90%). The median hospital stay was 1 day (range 1–3 days). Table 3 displays the surgical and postoperative outcomes obtained.

**Table 3:** Surgical and postoperative outcomes

Surgical and postoperative outcomes	Median (range)	Percentage
Operative time (min)	124.5 (102–198)	–
Conversion to laparoscopy	1	10%
Postoperative complications	0	0%
Hospital stay (days)	1 (1–3)	–

Source: Data collected from digital clinical records of patients undergoing robotic retroperitoneal adrenalectomy at Hospital Clínic de Barcelona, during the period July 2023 to February 2024.

### Complications and other observations

Of the 10 patients, 5 (50%) experienced a complication classified as Grade I according to the Clavien–Dindo scale. One of these patients (10%) required conversion to laparoscopy due to an inflammatory process caused by pyelonephritis. No other significant postoperative complications were reported. Table 4 summarizes the complications and other recorded observations.

**Table 4:** Complications and other observations

Complications and other observations	Number of cases	Percentage
Postoperative complications		
None	9	90%
Conversion to laparoscopy due to inflammation	1	10%
Other observations		
Clavien–Dindo Complications Grade I	5	50%

Source: Data collected from digital clinical records of patients undergoing robotic retroperitoneal adrenalectomy at Hospital Clínic de Barcelona, during the period July 2023 to February 2024.

### Comorbidities

Regarding comorbidities, Table 5 presents the comorbidities recorded in the patients.

**Table 5:** Comorbidities

Comorbidities	Number of cases	Percentage
Arterial hypertension	10	100%
Diabetes mellitus	4	40%
Dyslipidemia	3	30%
Obstructive sleep apnea syndrome	1	10%

Source: Data collected from digital clinical records of patients undergoing robotic retroperitoneal adrenalectomy at Hospital Clínic de Barcelona, during the period July 2023 to February 2024.

## Discussion

RRA has emerged as a promising technique in adrenal gland surgery, offering significant advantages in terms of precision, three-dimensional visualization, and less invasiveness. Previous studies have examined the safety of robotic-assisted (RA) surgery, but the benefits of RA compared to laparoscopic-assisted (LA) surgery remain under discussion [7, 8].

Recent research indicates no significant differences between robotic posterior retroperitoneoscopic adrenalectomy (RPRA) and posterior retroperitoneoscopic adrenalectomy (PRA) regarding estimated blood loss, postoperative pain, complications, or conversion rates [9]. However, although the robotic retroperitoneal technique had a longer operative time [9, 10], RA proved to be superior to LA in reducing hospital length of stay for pheochromocytoma.

The growing use of robotic-assisted surgery has raised ongoing debates about its cost-effectiveness compared to traditional techniques. Cost is a major factor when considering robotic surgery, with studies showing that it can be 1.2 to 3.2 times more expensive than laparoscopy [11]. This higher expense places a considerable strain on our healthcare infrastructure. Therefore, patient selection for robotic procedures should carefully weigh not only technical feasibility and clinical outcomes but also the broader economic and societal costs associated with the robotic approach.

In our study, we analysed the first 10 consecutive cases of RRA using the RAS Hugo™ platform at the Hospital Clínic of Barcelona. The results obtained were compared with other previous studies to contextualize our observations within the existing literature.

### Demographic comparison

**Gender:** In our study, 60% of the patients were men and 40% were women. In the 20-year experience study on videolaparoscopic adrenalectomy, 66.4% of the patients were women and 33.6% were men, indicating a higher proportion of women undergoing this type of surgery in their cohort. In the case report of a giant adrenal incidentaloma treatment, the patient was a man, which is not representative for a broader demographic comparison [12, 13].

**Age:** The median age in our study was 58 years (range: 50–73). In the 20-year experience study, the mean age was 46.7 years (range: 9–81), suggesting a slightly younger population with a broader age range, but close to our median age [12].

**Body Mass Index:** Our study reported a mean BMI of 28.5 kg/m^2^ (range: 21–36), which aligns with the 20-year study results where the average BMI was 27.44 kg/m^2^, with a range of 17–49 kg/m^2^ [12].

### Perioperative outcomes

In the initial study made by Barber et al., the mean operative time was 214.8 min [4]. However, the median operative time in our study was 124.5 min (range: 102–198), comparable to the median operative time of 144 min reported in the 20-year experience study on videolaparoscopic adrenalectomy conducted at the Hospital de Clínicas de Porto Alegre [12]. This similarity suggests that robotic surgery can be as efficient, if not more so, than traditional videolaparoscopic techniques regarding operative duration.

In a 2012 study by Ağcaoğlu et al., 31 cases of laparoscopic PRA were compared with 31 cases of RRA. They found no significant differences between the groups in terms of tumor size, blood loss, hospital stay, and skin-to-skin surgery times. However, after an initial learning curve of 10 cases, the operative times were significantly shorter in the RRA group (139 vs. 167 minutes, *p* = 0.046) [14].

Similarly, in a 2019 study by Kim et al., 169 patients underwent laparoscopic PRA and 61 underwent RRA, showing differences in the mean operation time, with laparoscopic PRA averaging 117 min and RRA averaging 142 min (*p* = 0.006) [15].

These findings indicate that while robotic surgery may require a longer learning curve, it has the potential to achieve operative times comparable to or shorter than traditional laparoscopic methods.

### Conversion rate

In our study, only one patient (10%) required conversion to laparoscopy due to inflammation caused by pyelonephritis. This rate is similar to the conversion rate of 8.2% (12 cases) reported in the videolaparoscopic adrenalectomy study [13]. This difference may be attributed to the technical advantages of robotic surgery, including better visualization and maneuverability, reducing the need for conversions.

### Hospital stay

The median hospital stay in our study was 1 day (range: 1–3), significantly shorter than the median of 4.5 days reported in the 20-year experience study [12]. This difference may reflect faster recovery and fewer postoperative complications with the use of robotic technology with a posterior retroperitoneal approach.

### Complications

No significant complications were reported in our study, except for one case of conversion to laparoscopy. In contrast, the reference study reported a 22.5% complication rate, with 10.9% intraoperative and 11.6% postoperative complications. In addition, the case report of giant adrenal incidentaloma treatment also indicated postoperative complications, including bleeding that was successfully managed. The lower complication rate in our study may be due to the improved precision and control offered by robotic surgery [13].

### Comparison of clinical outcomes

It has been shown that, once beyond the learning curve, the robotic approach shortens the operative time and causes less postoperative pain when compared to its laparoscopic counterpart [16]. In the research by Almagro et al., the use of robotic surgery for treating giant adrenal masses in patients with a history of laparotomy highlighted its safety and efficacy even in complex scenarios [13]. Similarly, our study demonstrated that RRA is a feasible and safe technique in selected patients, with no significant complications.

### Benefits and challenges

The robotic technique offers clear benefits in terms of superior three-dimensional visualization and greater maneuverability in confined spaces, facilitating the surgeon’s dissection maneuvers. However, one disadvantage mentioned in the literature is the limited workspace and lack of clear anatomical references in the posterior retroperitoneal access [17]. Despite these challenges, our study did not report significant problems related to these aspects, suggesting that the surgical team’s experience and familiarity with the technique are critical factors for success.

### Recent advances and comparative outcomes in robotic adrenal surgery

The study by Raffaelli et al. [18] reported the first five cases of lateral transabdominal adrenalectomy using the Hugo™ RAS system. While their work confirmed the technical feasibility of this new platform with no conversions or major complications and a median console time of 55 min, it employed the transabdominal route, involving intra-abdominal organ mobilization and a median hospital stay of 2 days. In contrast, our study applies Hugo™ RAS to the posterior retroperitoneoscopic approach, which avoids peritoneal entry and allowed a median hospital stay of 1 day, suggesting faster recovery and reduced surgical trauma in selected patients.

Further supporting our findings, Romero et al. compared robotic PRA to lateral transabdominal adrenalectomy and found similar operative times and complication rates, but significantly lower estimated blood loss in the PRA group (5 mL vs. 10 mL; *p* =0.001), underscoring a key intraoperative advantage of the posterior approach [19].

A recent meta-analysis by Esposito et al., which included 28 studies, also highlighted that robotic adrenalectomy is associated with lower blood loss, fewer conversions, and shorter hospital stays than laparoscopic adrenalectomy, though with higher associated costs. This reinforces the clinical benefits of robotic platforms while acknowledging the economic debate [20].

Innovative techniques, such as the tunneling approach introduced by Zhang et al. [21], and the renal-rotation technique described by Xue et al. for large adrenal tumors [22] show promise in enhancing the retroperitoneal route. These methods, especially when integrated with robotic systems like Hugo™, could further improve surgical access and safety in challenging adrenalectomies.

In terms of cost and efficiency, a study in surgical endoscopy found that retroperitoneoscopic adrenalectomy was more profitable and cost-effective than the laparoscopic transperitoneal approach, aligning with our observation that the shorter operative time and hospital stay in robotic PRA may contribute to favorable resource utilization [23].

## Conclusions

Our findings suggest that RRA is a viable and safe alternative to traditional laparoscopic techniques, with potential advantages in terms of shorter operative time, shorter hospital stay, and comparatively lower complication rates. Robotic technology can provide significant benefits in minimally invasive surgery, especially in complex cases or patients with previous surgical histories.

These results are aligned with the existing literature, although additional studies with a larger number of patients are needed to confirm these findings and fully evaluate the advantages and limitations of robotic surgery in adrenalectomy. The adoption of this technology requires investment in training and equipment, but the potential benefits for patients may justify these costs.

Our research has explored the outcomes and clinical implications of RRA in our patient cohort, representing our initial experience with this innovative surgical technique. Through our analysis, we have identified several key points that warrant attention and reflection.

1. **Safety and efficacy of RRA:** Our findings support the safety and efficacy of RRA in managing adrenal pathologies. The low rate of perioperative complications and the absence of mortality in our cohort reflect the feasibility of this technique as a viable surgical option for patients with adrenal diseases.
2. **Benefits of the retroperitoneal technique:** The retroperitoneal nature of RRA offers several advantages over transabdominal approaches, including less manipulation of intra-abdominal organs and potentially faster recovery times. Our results support these benefits, with shorter hospital stays and earlier postoperative recovery compared to similar transabdominal approaches.
3. **Technical challenges and learning curve:** However, it is important to recognize the technical challenges associated with RRA and the learning curve required for mastering this technique. Our initial results may reflect an early learning phase, and greater experience may be needed to further optimize surgical outcomes and reduce operative time.
4. **Oncological and functional considerations:** Robotic adrenalectomy presents unique considerations in terms of oncology and preserved adrenal function. While our research focuses on the initial experience, it is crucial to evaluate long-term outcomes in terms of oncological control and postoperative adrenal function to fully understand the impact of RRA on these aspects.
5. **Implications for clinical practice:** Our findings have important implications for clinical practice, highlighting the feasibility and potential benefits of RRA in managing adrenal diseases. However, more prospective studies and long-term follow-up are needed to validate our results and provide more robust clinical guidance in this area.

## Data Availability

The data supporting the conclusions of this study, including detailed patient information, procedure outcomes, and other relevant data, are available upon request from the corresponding author.
